# Supplementary material for: Near-surface magma flow instability drives cyclic lava fountaining at Fagradalsfjall, Iceland
Source: Nat Commun. 2023 Nov 7;14:6810. doi: 10.1038/s41467-023-42569-9 (PMC10630439; doi:10.1038/s41467-023-42569-9)
Supplement: Supplementary file 1 — Supplementary Information [file 41467_2023_42569_MOESM1_ESM.pdf]

Supplementary Materials for "Near-surface magma flow instability drives cyclic lava fountaining at Fagradalsfjall, Iceland"

Samuel Scott[1], Melissa Pfeffer[2], Clive Oppenheimer[3], Enikö Bali[1], Oliver D. Lamb[4, 5], Talfan Barnie[2], Andrew W. Woods[6], Rikey Kjartansdóttir[1], Andri Stefánsson[1]

1: Institute of Earth Sciences, University of Iceland, Sturlugata 7, 101 Reykjavík, Iceland

2: Icelandic Meteorological Office, Bústaðavegur 7-9, 105 Reykjavík, Iceland

3: Department of Geography, University of Cambridge, Downing Place, Cambridge CB2 3EN, UK

4: Department of Earth, Marine and Environmental Sciences, University of North Carolina at Chapel Hill, 104 South Road, Chapel Hill, NC 27599-3315, North Carolina, USA

5: Te Pū Ao | GNS Science, Wairakei Research Centre, 114 Karetoto Road, RD4, Taupō 3384, NZ

6: BP Institute, University of Cambridge, Cambridge CB3 0EZ, UK

This PDF file includes:

Figs. S1 to S6

Table S1

Caption for Movie S1

Other supplementary materials for this manuscript includes the following:

Movie S1 (.mp4)

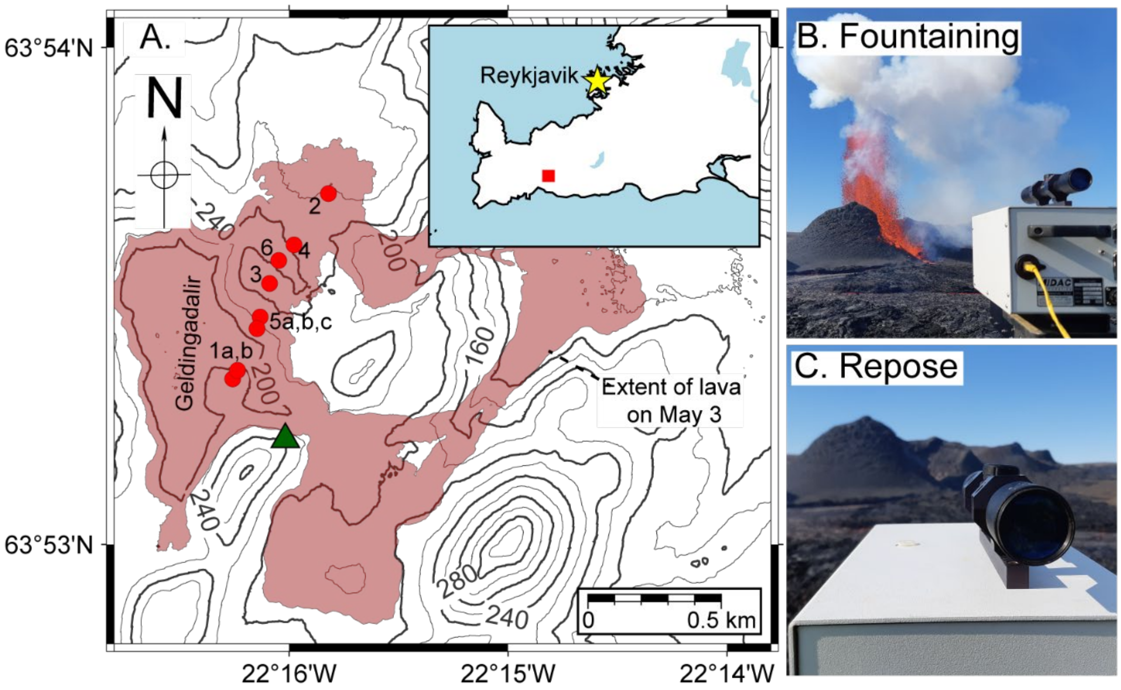

Figure S1. (a) Map of the Fagradalsfjall area showing the eruptive vents (red circles), the extent of the lava field on 3 May 2021 (red area), and observation point for open-path FTIR spectroscopy on 5 May (green triangle). Inset shows the location of Fagradalsfjall on the Reykjanes peninsula in SW Iceland. (b, c) Photos showing the FTIR spectrometer pointed at Vent 5 during fountaining and repose intervals, respectively.

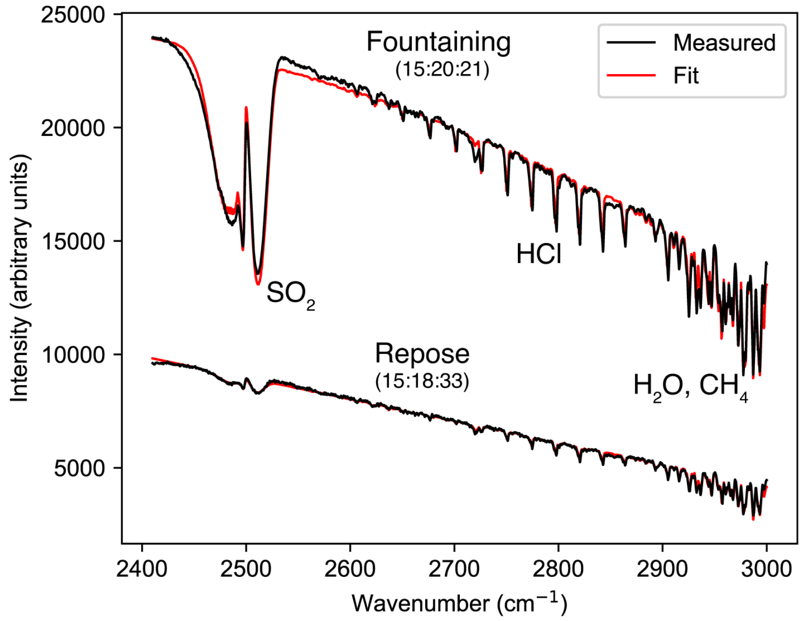

Figure S2. Examples of spectra collected during fountaining and repose (black lines). Red lines show modeled spectra using the software FTIR-FIT (Burton et al., 2007). Note higher volcanic gas abundances (and signal intensity) during fountaining (upper spectrum). Spectral features for SO_2, HCl, H_2O and CH_4 are indicated, the latter arising only from the atmospheric background.

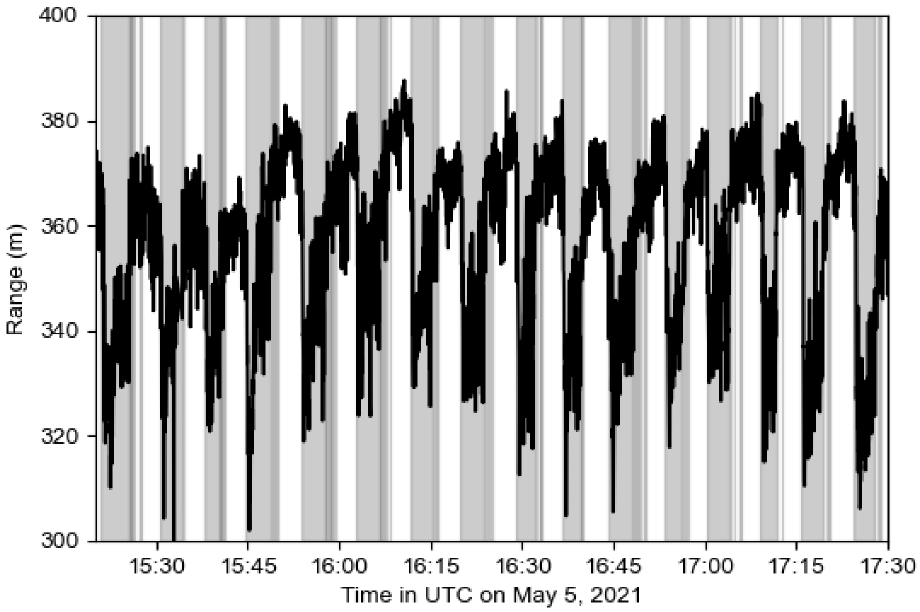

Figure S3. Computed range (distance to IR source) based on methane abundance and an atmospheric methane mixing ratio of 1.93 ppmv. Atmospheric pressure and temperature are taken as 985 hPa and 276 K, respectively (based on readings from nearby meteorological monitoring station). Range decreases during the fountaining intervals (shown in grey) as the distance between the spectrometer and the IR source shortens due to the erupting lava. Range increases during the repose periods when the IR source becomes the far side of the vent.

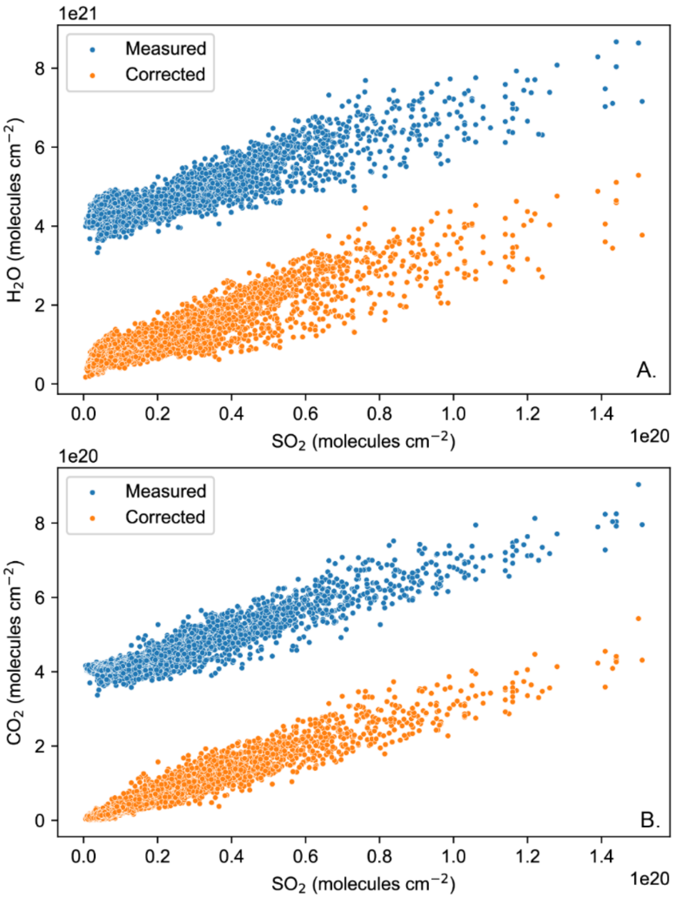

Figure S4. Raw (blue) and corrected (orange) column amounts for a) H_2O and b) CO_2, plotted against SO_2.

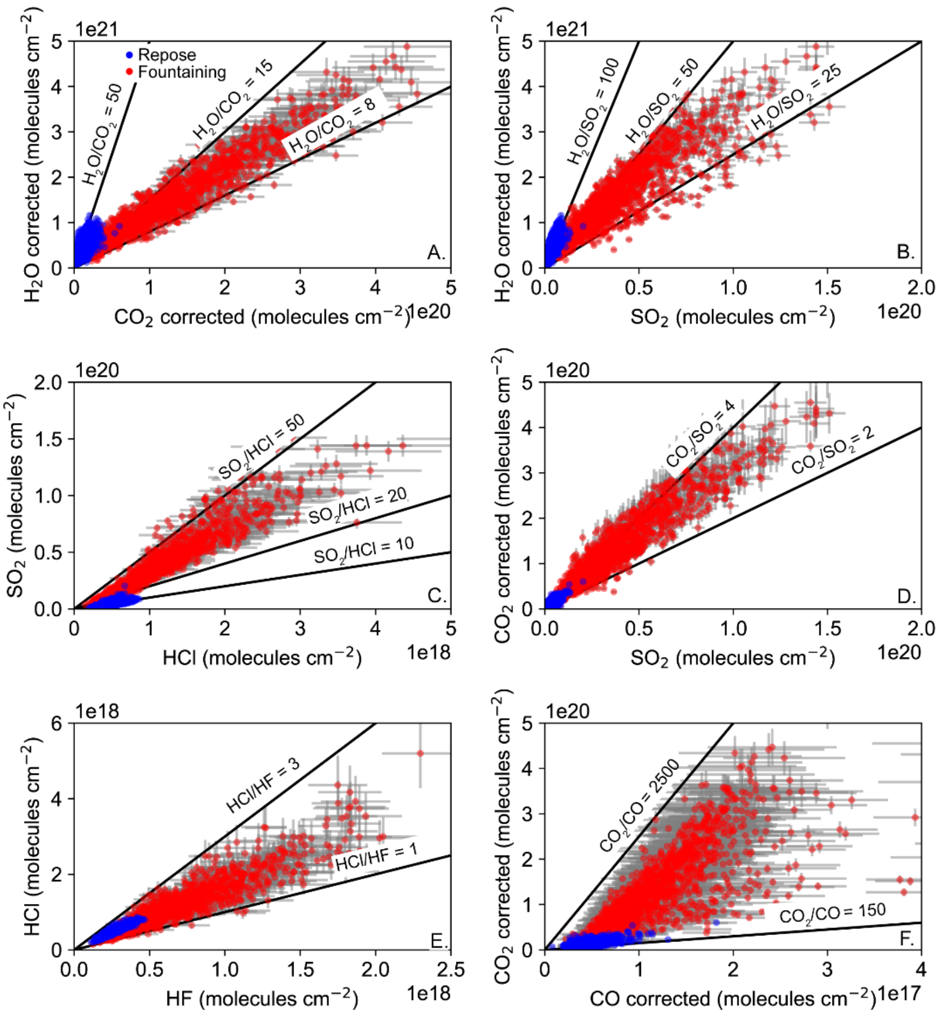

Figure S5. Scatter plots of column amounts for pairs of gases to determine the ratios. Red circles represent measurements during fountaining and blue during repose. H_2O, CO_2, and CO have been corrected for atmospheric background. Analytical errors associated with retrievals of column amounts from measured spectra (calculated by FTIR-FIT) are shown with grey bars. Note that the errors for many of the repose measurements with low gas contents are less than the symbol size. A) H_2O vs. CO_2, B) H_2O vs. SO_2, C) SO_2 vs. HCl, D) CO_2 vs SO_2, E) HCl vs HF, F) CO_2 vs. CO.

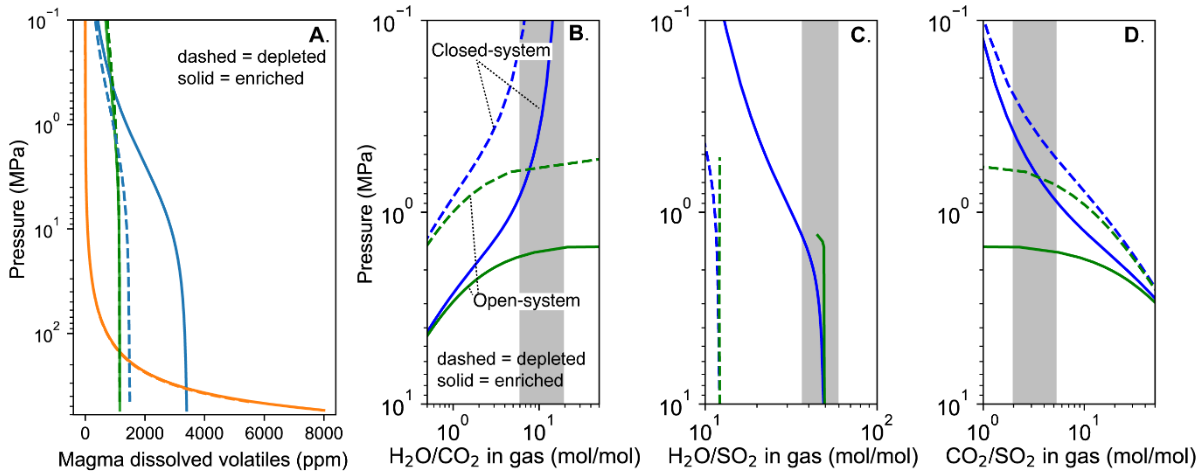

Figure S6. Sensitivity analysis for degassing calculations of the Fagradalsfjall melt, comparing depleted melt (dashed lines) and enriched melt (solid lines). Initial volatile compositions for depleted and enriched melts given in text – the depleted melt is distinguished by a lower H_2O content. **a** Dissolved CO_2, H_2O, and S concentrations in the melt as a function of pressure. **b** H_2O/CO_2, **c** H_2O/SO_2, and **d**. CO_2/SO_2 after a stage of gas loss at 100 MPa (see text). Closed- and open-system degassing after gas loss at 100 MPa shown with blue and green lines, respectively.

Table S1. Volatile contents of the more enriched melt derived on the basis of trace element proxies.

Eruption day	Ba (ppm)	Nb (ppm)	Ce (ppm)	Dy (ppm)	CO_2 (ppm) Ba*133	CO_2 (ppm) Nb*505	H_2O (ppm) Ce*180	S (ppm) Dy*247	S (ppm) Dy*350
36	82.8	13.1	19.6	3.7	11014	6625	3528	922	1306
37	79.8	15.7	18.8	3.3	10620	7954	3390	812	1150
37	80.8	12.1	18.8	3.5	10740	6127	3389	858	1216
43	77.6	9.2	18.3	3.5	10326	4652	3297	861	1220
**6th of May day 48**	**72.9**	**9.2**	**17.3**	**3.5**	**9701**	**4662**	**3107**	**853**	**1209**
53	85.7	11.0	19.9	3.5	11392	5539	3577	872	1236

Movie S1. Time-lapse movie of RÚV camera footage between 14:59–15:59 GMT on 5 May 2021, during the FTIR measurement period. Yellow line in the top panel shows the fountain 'height'. The bottom panel shows the height of the fountain as a function of time. Overflows from the lava lake are visible during the fountaining. This is followed by draining and decrease in the height of the lava lake by ~10 m in between the fountaining events.
