# Supplementary material for: Near-surface magma flow instability drives cyclic lava fountaining at Fagradalsfjall, Iceland
Source: Nat Commun. 2023 Nov 7;14:6810. doi: 10.1038/s41467-023-42569-9 (PMC10630439; doi:10.1038/s41467-023-42569-9)
Supplement: Supplementary file 3 — Description of Additional Supplementary Files [file 41467_2023_42569_MOESM3_ESM.pdf]

Description of Additional Supplementary Files

File Name: Supplementary Movie 1
Description: Time-lapse movie of RÚV camera footage between 14:59–15:59 GMT on 5 May 2021, during the FTIR measurement period. Yellow line in the top panel shows the fountain 'height'. The bottom panel shows the height of the fountain as a function of time. Overflows from the lava lake are visible during the fountaining. This is followed by draining and decrease in the height of the lava lake by ~10 m in between the fountaining events
